# Reviews

**Published:** 1880-06

**Authors:** 


### REVIEWS.

---

**A Treatise on Foreign Bodies in Surgical Practice.** By ALFRED PAULET, M. D., Adjutant Surgeon-major, Inspector of the School for Military Medicine, at Val-de-Grace. New York: William Wood & Co. 1880.

This work, one of Wood's Library of Standard Medical Authors, in two volumes, is quite a novelty, in so far as nothing analogous has ever been published. We have here a large and complete work, gathering together what formerly could only be found with difficulty in journals, monographs, or scattered through the larger surgical works. The author considers in this work only the foreign bodies of the natural passages, while he promises later to treat the two other groups, foreign bodies, which enter the economy by "effraction," or are fixed to the surface of the parts. The book will be of great value to every physician, the more so as foreign bodies are an accident, which every physician occasionally is called upon to treat. M.

---

**Headaches; their nature, causes and treatment.** By WILLIAM HENRY DAY, M. D, Member of the Royal College of Physicians, of London, Physician to the Samaritan Hospital for women and children. Third Edition with Illustrations. Philadelphia: Lindsay & Blakiston.

The wide prevalence of headache, under a great variety of forms and conditions, and the unsatisfactory results of medical treatment, will attract to this work the attention of the profession. The views advanced by the author are the result of notes and observations carefully recorded, and extending over a period of many years. The author also strives to make the work practically useful, by adopting the division of headache into several varieties, showing the differential diagnosis in the anæmic forms and the hyperæmic, the characteristic features of sympathetic, of bilious, or dyspeptic, nervous, toxæmic, gouty, rheumatic headaches, &c., &c. A careful examination of the book gives a most favorable impression of its merits and of its practical utility, in the investigation of Cephalalgia. We recommend it to such of the profession, who, if not victims of the disease and the conditions of which it treats, are frequently called upon to relieve sufferings due to this cause. L.

---

**The Principles and Practice of Gynæcology.** By THOMAS ADDIS EMMETT, M. D., Surgeon to the Woman's Hospital, &c. With one hundred and thirty illustrations. Philadelphia: Henry C. Lea. 1879.

The author announces in the preface that this work is essentially a clinical digest, and includes the results of individual experience for twenty-five years in the treatment of diseases of women. In addition to opportunities offered in a very extensive private practice, Dr. Emmett summarizes in this work his experience in the Woman's Hospital, of which for many years he has been the recognized surgeon-in-chief. From such a wide field the accomplished author has enjoyed rare facilities, through which he has demonstrated the ability and skill which has placed his name among the most distinguished gynæcologists of the present day.

There is much to commend in every chapter, and in attempting a review, the writer especially desires to direct attention to the philosophical manner in which every subject is treated. Dr. Emmett deals in facts and bases his conclusions upon fundamental principles. He avoids useless theories, unfolds his reasons in clear and concise language, condenses in tabular form important data in regard to various conditions to which special study and observation have been given, and as the result furnishes the most valuable and complete work ever offered to the profession in the special department in which he has labored.

The subjects treated in this volume comprise almost every condition met with in gynæological practice.

The chapters devoted to Uterine Displacements show clearly the author's ingenuity in dealing with a most difficult condition. His views on the etiology and treatment of flexures and versions, the objects, uses and adjusting of pessaries, the influence of menstruation in flexures, &c., are singularly clear and practical, while the surgical measures recommended are based upon sound and well-established principles.

Dr. Emmett lays stress upon laceration of the cervix uteri as a condition too frequently overlooked, except by the specialist. He justly states that "its importance cannot be over-estimated, since at least one-half of the ailments, among those who have borne children, are to be attributed to this cause."

The extensive experience of the author in vesico- and recto-vaginal fistulas gives special interest to the chapters which are devoted to this accident of the puerperal state. Probably no other surgeon operates with so much skill and with such surprising results. He embodies his views very fully in this work, devoting ample space to this condition, and shows his own plan of operating in a very clear and lucid manner.

These few selections from the many subjects treated in this valuable work will suffice to show how well Dr. Emmett has performed the duty of furnishing the profession, from his abundant storehouse of experience and knowledge, a guide to the study and treatment of diseases of women.

We are proud that American skill and ability is thus utilized in an honest effort to advance the interests of an important department of medical science.

L.

---

**A Practical Treatise on Sea-sickness; its Symptoms, Nature and Treatment.** By GEORGE M. BEARD, A. M., M. D. New York: E. B. Treat, 707 Broadway. 1880.

Dr. Beard presents a very excellent monograph upon a disease which has not been studied as much as, in these days of foreign travel, its importance demands. He affirms that sea-sickness is a functional disease of the central nervous system, and the treatment proposed is based upon the etiology he endeavors to establish. The author has given so much study and thought to nervous diseases, that whatever issues from his pen is entitled to respectful consideration from the profession. L.

---

**Sore Throat, its Nature. Varieties and Treatment; including the connection between affections of the throat and other Diseases.** By PROSPER JAMES, M. D., Lecturer on Materia Medica and Therapeutics at the London Hospital, etc. Fourth edition. Illustrated with hand-colored plates. Philadelphia: Lindsay & Blakiston, 1880.

This is the fourth edition of a well-known little work, a fact which shows the favor it has found with the profession. Some changes have been made, and four new chapters, about syphilitic sore throat, affections of the naso-pharynx, connection of sore throat with affections of the nose and the ear, affections of the œsophagus, have been added. The work is a handy book for reference, and is well worth reading. M.

